# Elimination of *Simulium neavei*–Transmitted Onchocerciasis in Wambabya–Rwamarongo Focus of Western Uganda

**DOI:** 10.4269/ajtmh.20-0195

**Published:** 2020-06-22

**Authors:** Moses N. Katabarwa, Peace Habomugisha, Annet Khainza, David Oguttu, Edson Byamukama, James Katamanywa, Thomson Isingooma, Fredrick Bwenume, Christine Nahabwe, Monica Ngabirano, Paul Akampurira, Lauri Bernard, Thomas R. Unnasch, Frank Richards

**Affiliations:** 1 The Carter Center, Atlanta, Georgia;; 2 The Carter Center, Kampala, Uganda;; 3 Vector Control Division, Ministry of Health, Kampala, Uganda;; 4 Vector Control, Kabarole, Uganda;; 5 Hoima District Health Service, Hoima, Uganda;; 6 Center for Global Health Infectious Disease Research, University of South Florida, Tampa, Florida

## Abstract

Wambabya–Rwamarongo onchocerciasis focus is one of the eight foci Uganda verified using the WHO verification guidelines. The approach for elimination was twice yearly treatment with ivermectin for every round, treating at least 90% of all the eligible population. This was in combination with vector elimination using Abate^®^ (BASF SE, Limburgerhof, Germany) since elimination nationwide policy was launched. From 2008 to 2013, the program distributed ivermectin with a mean treatment coverage of the ultimate treatment goal (UTG) or eligible population of 91.2%, with a range of 85–96%. In 2009, vector elimination based on ground larviciding had a dramatic impact on the *Simulium* vectors, as the last fly was observed in October 2009. No more *Simulium* vectors were observed during a period of at least 7 years, including the 3-year posttreatment surveillance (PTS) until the focus was reclassified as eliminated in August 2017. During the PTS period, none of the 10,578 trapped crabs were found infested with the aquatic stages of the vector. The last infested crab was observed in March 2010, and for at least 7 years, no infested crabs were observed. Serological surveys showed that of 2,978 young children examined in 2013, only one was OV16 positive (0.0%; 95% CI: 0–0.21). In 2017, after the PTS period, all 3,079 young children examined were negative for OV16 (95% CI: 0–0.16). Therefore, entomological and serological results provided evidence that resulted in the reclassification of Wambabya–Rwamarongo focus from “transmission interrupted” to “transmission eliminated” with no possibility of recrudescence.

## INTRODUCTION

Wambabya–Rwamarongo onchocerciasis focus is one of the 17 onchocerciasis foci in Uganda. The focus was shared between Hoima and Kikuube (a new district that was recently curved out of Hoima) districts of Western Uganda. It is believed to have been part of the Budongo onchocerciasis focus, which is to the northeast involving the districts of Buliisa, Hoima, and Masindi. *Simulium neavei*, the only vector of onchocerciasis in this focus, breeds where the rivers and streams are fast flowing. *Simulium neavei* exists in a phoretic association with freshwater crabs (*Potamonautes niloticus*). The larval stages of *S. neavei* attach to the crabs for transport and access to food. This vector requires shaded areas, thriving in and around the natural forests of Bujaawe and Wambabya in this focus.

Breeding of this vector was thought to extend from the Bugoma Forest Reserve to the southwest through to the Budongo Forest.^1,2^ Investigations carried out in the Bugoma Forest Reserve and the Wambabya–Rwamarongo area in 1967 did not find evidence for transmission of onchocerciasis.^2^ During that period, the Bugoma Forest Reserve and Wambabya–Rwamarongo areas were largely uninhabited, and only the Budongo area was a known onchocerciasis focus with 80% of the sawmill workers and forestry students infected.^2,3^ Later, the focus was included on a map showing the distribution of onchocerciasis by Prentice in 1974.^4^

Nine *S. neavei*–transmitted onchocerciasis foci in Uganda were confirmed in 1979.^5,6^ These foci included Budongo, Bugoma, Bwindi, Kashoya–Kitomi, Itwara, Wambabya–Rwamarongo, West Nile, Imatong, and Mt. Elgon. However, since 1996, unpublished entomological and epidemiological surveys have not indicated the existence of onchocerciasis transmission in Bugoma and Imatong areas. To date, Bugoma is still a protected forest reserve and, therefore, uninhabited. That is why Bugoma and Imatong foci are not reflected in the recent official government reports and published articles.^7-^^9^

In 1989, onchocerciasis was declared a disease of public health importance in Budongo and Wambabya–Rwamarongo areas of Masindi and Hoima districts, and distribution of an annual dose of ivermectin per person, provided free by Merck & Co (Kenilworth, NJ), commenced. The distribution was supported by Sightsavers through the Uganda Foundation for the Blind with additional support from AVSI (Italy) from 2003 to 2006. Later, Sightsavers assumed directed support status for the Wambabya–Rwamarongo focus. Under the nationwide Uganda onchocerciasis elimination policy launched in 2007, the Wambabya–Rwamarongo onchocerciasis focus began receiving twice per year treatment with ivermectin complemented with vector elimination.^9^ The Carter Center provided support for vector elimination using donated Abate^®^ (trade name, Temephos™ 500 EC), an environmentally friendly organophosphate larvicide^8,10^ from Badische Anilin und Soda Fabrik (BASF). Although the disease was not blinding affected persons, they experienced debilitating skin disease as described in.^11–13^

## MASS TREATMENT WITH IVERMECTIN

### Control era (1989–2006).

Unpublished reports indicate that annual mass treatment with ivermectin donated by Merck and Co., Inc began in Wambabya–Rwamarongo in 1989. No baseline survey on the status of infection was conducted before treatment began.

### Elimination era (2007–2016).

In early 2007, Uganda launched a nationwide policy for elimination of onchocerciasis (Katabarwa et al., 2018).^9^ The strategy was to treat all eligible persons in a focus twice yearly and attain at least 90% of all the eligible population in every onchocerciasis-endemic focus annually and complement it with vector elimination/control where feasible, through ground larviciding with Abate^®^. In 2013, transmission in the focus was declared interrupted, and the focus was moved to a 3-year posttreatment surveillance (PTS) period.

### Uganda onchocerciasis elimination expert committee (UOEEAC).

**Figure 1.**
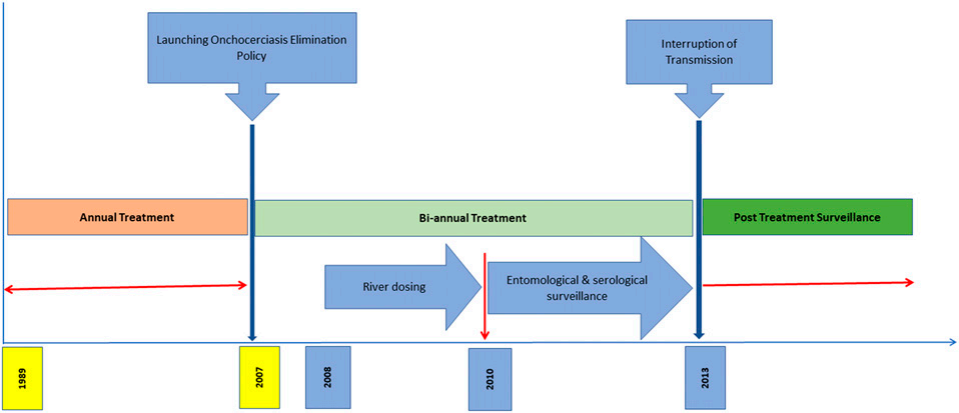
Timeline for elimination of onchocerciasis in the Wambabya–Rwamarongo focus.

In 2017, the UOEEAC declared the Wambabya–Rwamarongo onchocerciasis focus eliminated.^9^ The present article reports on the treatment and assessment activities conducted in support of the conclusion that *Onchocerca volvulus* transmission was eliminated in the Wambabya–Rwamarongo focus in 2017. The timeline for interventions toward elimination of onchocerciasis is illustrated in Figure 1.

## METHODS

### Study area.

**Figure 2.**
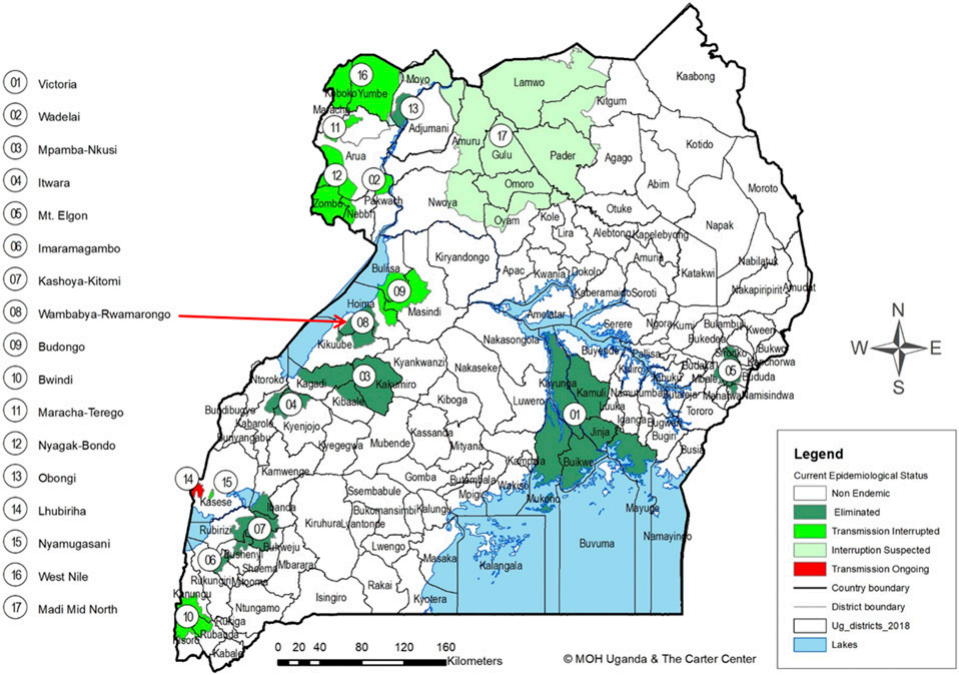
Map of Uganda onchocerciasis foci showing the location of the Wambabya–Rwamarongo focus.

The Wambabya–Rwamarongo onchocerciasis focus in Western Uganda (Figure 2) is traversed by Wambabya and Rwamarongo river systems which originate from Kyabigambire subcounty. The tributaries for the River Wambabya include Itobya and Ntalya. Other small and fast-flowing streams in the focus do not support vector breeding. The focus covers the subcounties of Biseruka, Bugambe, Kigorobya, Kiziranfumbi, and Kitoba. The upper reaches of the Wambabya–Rwamarongo focus toward the Budongo focus are covered by extensive papyrus swamps. There are also sugar plantations that apply agrochemicals, resulting in unfavorable conditions for *S. neavei* breeding. Therefore, the stretch between Wambabya–Rwamarongo and Budongo of at least eight kilometers acts as a “buffer” zone between these two transmission zones or foci.

**Figure 3.**
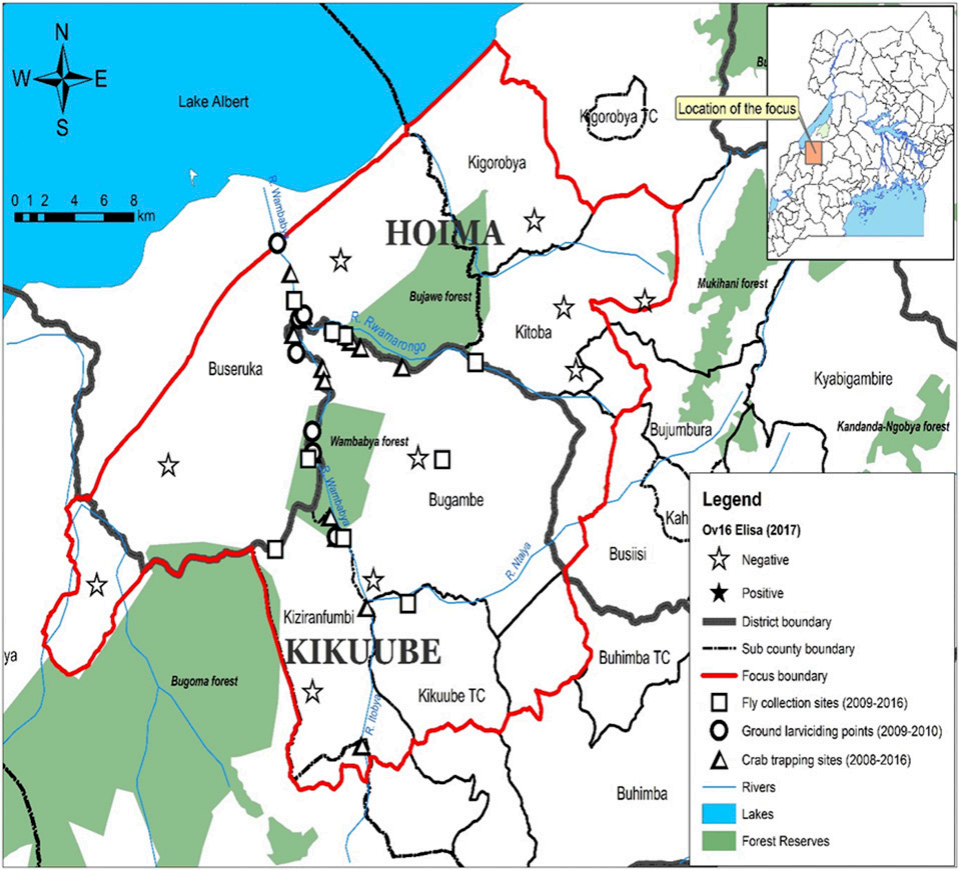
Map of the Wambabya–Rwamarongo onchocerciasis focus showing OV16 results, fly collection sites, and ground larviciding points.

A transmission zone as defined by the WHO is a geographical area where transmission of *O. volvulus* occurs by locally breeding vectors and which can be regarded as a natural ecological and epidemiological unit for interventions.^14^ The buffer zone was defined as at least a 20-km limit where *Simulium damnosum*–infected flies cannot cross from one transmission zone to another.^15^ Because the only vector in these foci was *S. neavei* which exhibits a flight range less than 6 kms,^16^ a buffer zone of at least 8 kms between the Wambabya–Rwamarongo focus was sufficient to prevent infected vectors from crossing Budongo. Moreover, when there is no shade, the flight distance for *S. neavei* is drastically reduced, as the flies avoid sunny or hot open areas.^17^ Therefore, Wambabya–Rwamarongo was an isolated focus that extended along the escarpment of the Western Rift Valley and Lake Albert, covering about 1,392 km^2^ (Figure 3).

The total population in the Wambabya–Rwamarongo focus was about 77,000 people. A report to the United Nations Development Programme/World Bank/WHO Special Programme for Research and Training in Tropical Diseases, 1996, concluded that the area should be surveyed with a view of implementing vector elimination operations.^7^ This report resulted in the recommendation of vector elimination in the Wambabya–Rwamarongo focus.^18^ When the Uganda government declared a nationwide elimination of onchocerciasis, vector elimination along with twice per year treatment with ivermectin was implemented in this focus.

### Mass treatment (1989–2013).

Annual treatment commenced in 1989, although treatment records up to 1995 were not available. Additional support from The Carter Center for twice per year treatment commenced in 2007, and by the end of 2013, transmission of *O. volvulus* in this focus was declared interrupted by the UOEEAC, and treatment was halted. The focus was moved to a 3-year posttreatment surveillance based on the recommendation of the UOEEAC meeting in August 2013. When refining of the delineation was carried out in 2007, additional communities classified as “hypoendemic” for onchocerciasis were included in the mass treatment with ivermectin plan.

### Entomological activities (2008–2016).

#### River prospections

Entomological activities in the Wambabya–Rwamarongo focus began in October 2008 with river prospections. This involved walking along the river systems, trapping crabs, and collecting and identifying black flies present. The objective of determining if onchocerciasis vectors were present and if so where best yielding collection sites and crabs were to use for periodic monitoring of the status of transmission. The information obtained also helps locate nine ground larviciding/dosing sites (six on the R. Wambabya and three on the R. Rwamarongo) with 11 crab monitoring/trapping sites and 12 fly collection sites. Vector elimination was the objective.

#### Ground larviciding

Abate trials in the focus were conducted in June and July 2009, and actual ground larviciding commenced in September 2009 ceasing in October 2010. Six dosing points were established on the R. Wambabya and three on the R. Rwamarongo. Larviciding continued for the next 12 months, initially at 4-week intervals for 6 months and 8-week intervals thereafter.^19^ Abate^®^ was applied at a rate of 0.2–0.4 mg/L to reach a concentration of 0.1–0.3 ppm. The insecticide was premixed in a 15-L knapsack sprayer and applied for 30 minutes at established dosing points. The impact of the larvicide on *S. neavei* immature stages was assessed 2 days after every larviciding event. Entomological surveillance continued after all interventions were halted through to the time when the focus was declared eliminated. When larviciding was stopped, surveillance was achieved through monitoring of crab infestation and *S. neavei* collection. Entomological surveillance involved monitoring of crab infestation and verifying the presence or absence of flies for 3 years. If no adult flies or infested crabs were observed for 3 years, then transmission interruption of *O. volvulus* could be declared, and the focus moved to 3 years of PTS—Uganda, 2011.^8^

### Assessments during elimination interventions.

#### Crab trapping

Crab trapping was applied using a funnel-shaped basket traps baited with fresh meat (Garms et al., 2009).^19^ The traps were placed in a hidden or covered position in the river along the banks to prevent light and encourage the crabs to enter them. This was carried out regularly every year through the intervention period. During the first 2 years, crab trapping was performed monthly from October 2008 to May in 2009 and then repeated in September 2009 through April 2010. Crab trapping was then carried out in the focus in June, August, and October 2010. From January 2011 to June 2013, it was carried out approximately every 2 months. The trapped crabs were examined, and the proportion infested with aquatic stages of *S. neavei* was determined. The objective for interruption of transmission was to have zero crab infestations throughout the focus for a minimum of 3 years.^8,20^

#### Fly collection

Fly collection was conducted monthly, and when no *S. neavei* was observed or collected for 3 years, transmission in the focus was considered interrupted (Uganda 2011). In Wambabya–Rwamarongo, fly collection began in January 2009 and continued through 2017.

Uganda’s guidelines state that interruption of transmission is attained when no flies or infested crabs have been observed for 3 years. In Wambabya–Rwamarongo, this type of assessment was conducted for at least three and a half years.^8,20^

#### Posttreatment surveillance (2014–2017)

During the 3-year and 5-month PTS period, crab trapping and assessing for aquatic stages of *S. neavei* attached to the freshwater crabs continued every quarter at all 11 crab-monitoring sites along Wambabya and Rwamarongo river systems from January 2014 to May 2017, when onchocerciasis was declared eliminated. Fly collection was also carried out at 12 fly collection sites during the PTS period (2014–2017). When fly elimination is the objective, progressive reduction of the fly population to zero was taken as an indicator of transmission interruption in a focus.

A total of 3,005 *Simulium* flies were collected and morphologically identified. Of this number collected, 43.1% were analyzed by PCR to determine their infectivity rate using the O-150 PCR protocol to detect *O*. *volvulus* DNA. Pool Screen^®^ software (Version 2.0; University of 238 Alabama, Birmingham, AL) was used to estimate the proportion of flies carrying infective stages of *O. volvulus* as well as the associated 95% CIs.^15^

### Epidemiological assessments.

#### Serology (OV16 ELISA) in children younger than 10 years (2008 versus 2013)

In 2008, dried blood spots (DBSs) taken from a total of 2,796 resident children younger than 10 years were examined. This survey was repeated in 2012 where DBS samples were obtained from 2,978 resident children of the same age. Because this focus was relatively small, the program aimed at collecting DBSs from all resident children. After 3 years PTS, another sample of 3,079 blood spots was collected in late 2016. Dried blood spot samples were proportionally assigned by parish population and subcounty to ensure that the samples were well distributed across the focus. Then, more samples were purposively collected in the “first-line” communities close to the Wambabya and Rwamarongo river systems where *Simulium* breeding was supported. The population figures were obtained from the household registers that were updated yearly. Sterile procedures as per the existing protocol were followed, and blood spots from every selected child younger than 10 years were collected on Whatman no. 2 filter paper (Sigma). The blood samples were dried, separated by sheets of paper, systematically packed, and stored in plastic bags that were placed in a cooler. On reaching the molecular laboratory at the Vector Control Division in Kampala, they were stored at 4°C before analysis. Sera were eluted from the dried spots and examined for the presence of OV-16 IgG4 antibodies by ELISA as per the standard protocol.^21^

### Ethical approval of the study.

The Emory Institutional Review Board (11,438) and the Ministry of Health of Uganda classified the assessment activities as periodical program performance assessment (non-research). This included parasitological, serological, and entomological assessments. Where individual community members were involved, consent was obtained from everyone involved, and verbal assent was obtained from the parents of young children. Participating communities were educated about the importance of assessments, and there were no repercussions for individuals who refused to participate. The *S. neavei* collectors were adults older than 20 years who had consented to participate in the activity. Opting out of the study if they so wished at any time without any repercussions was a prerogative of all participants.

## RESULTS

### Mass treatment with ivermectin.

**Figure 4.**
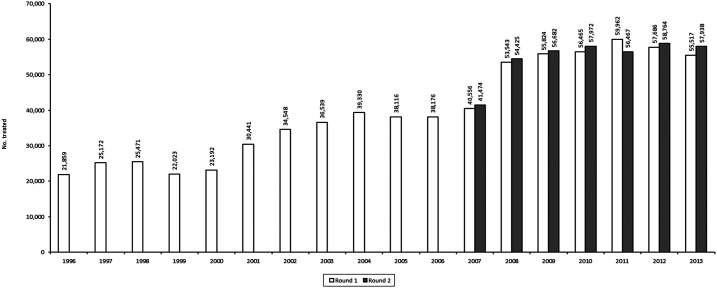
Number of persons treated with ivermectin from 1996 to 2013.

**Figure 5.**
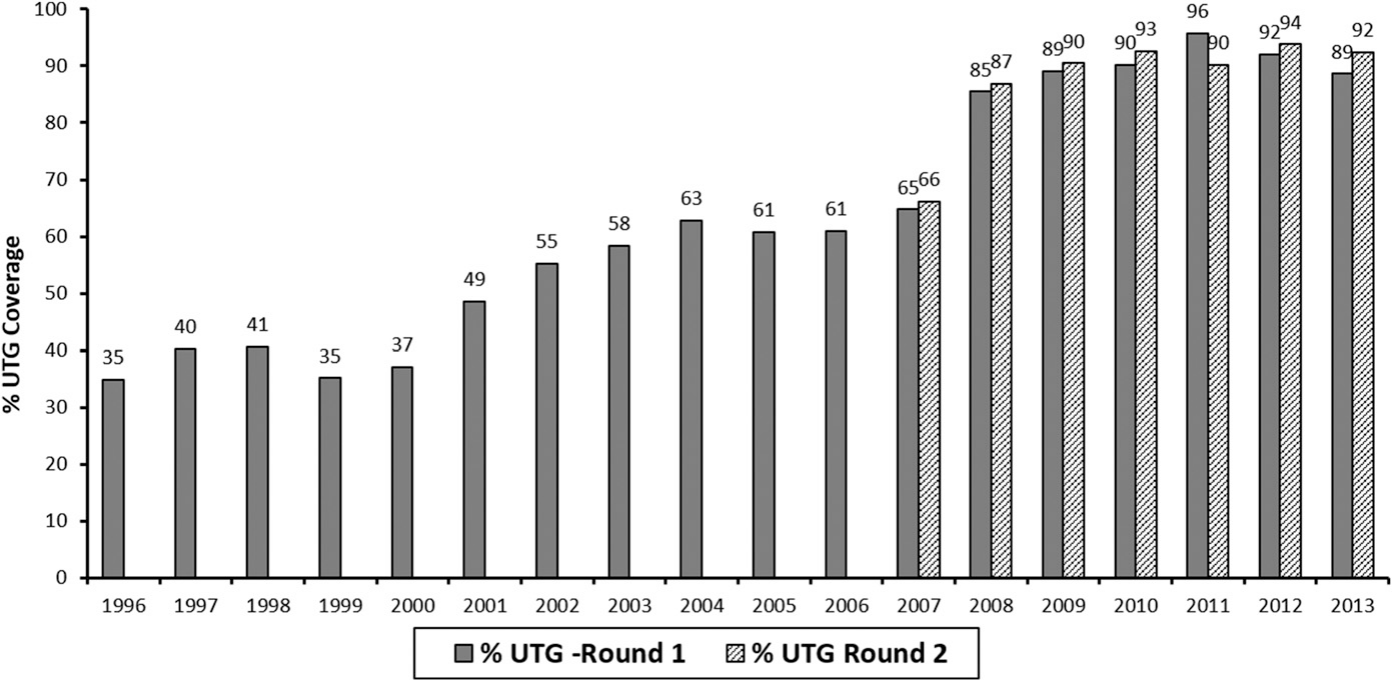
Percent of ultimate treatment goal (UTG) coverage (1996–2013).

The number of treatments and ultimate treatment goal (UTG) in this focus was below the desired level of at least 90% before the nationwide elimination policy was implemented in 2007 (Figures 4 and 5). The mean UTG coverage for the annual treatment was 50% for 12 years (1996–2008), with a range of 35–66%. From 2008 to 2013, the mean for 6 years of the twice per year UTG treatment was 91.2%, with a range of 215 85–96%.

### Entomological assessments.

#### *Simulium neavei* surveillance

**Figure 6.**
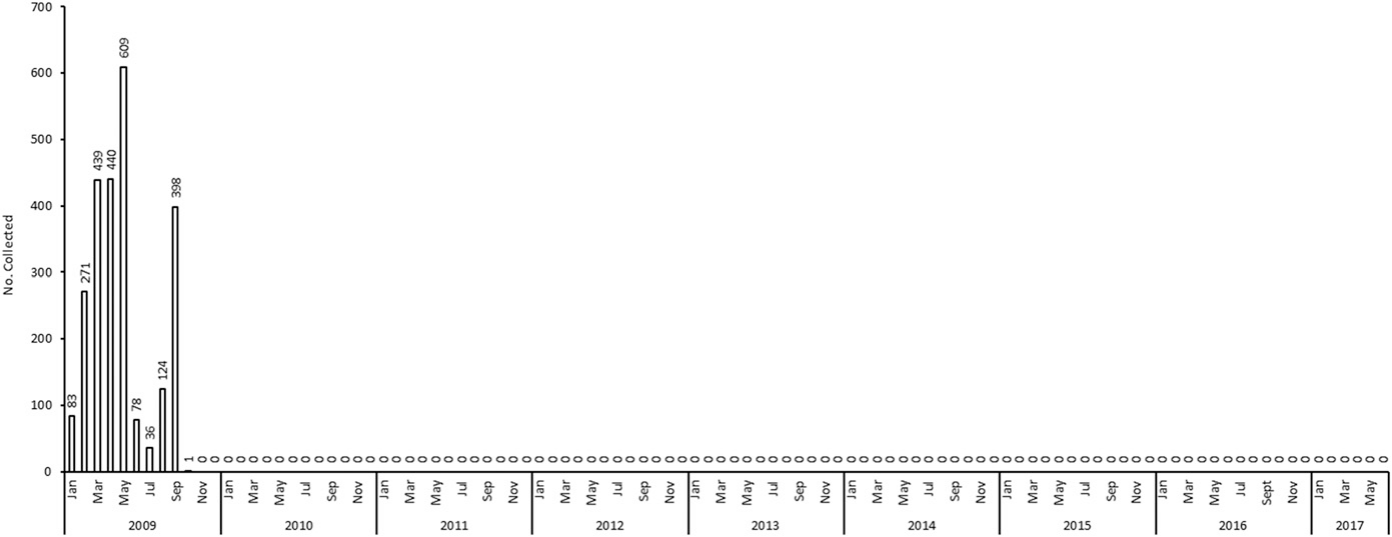
Trend of *Simulium neavei* elimination (2009–2017).

The last fly in the focus was observed in October 2009, and no *S. neavei* adult fly was observed for at least the seven subsequent years (Figure 6). A total of 3,005 flies were caught during the PTS period; all were morphologically identified as *Simulium ardesi*, a nonvector species of *Simulium* (Table 1). All the 1,296 (43.1%) flies were negative for the DNA of *O. volvulus*.

**Table 1:** Total number of *Simulium adersi* collected during the posttreatment surveillance period (2014–2017)

Year	Number of fly collection sites	Number of *Simulium neavei* collected	*Simulium adersi*
2014	8	0	550
2015	8	0	932
2016	8	0	986
2017	8	0	537
	Total	0	3,005

#### Crab infestation

**Figure 7.**
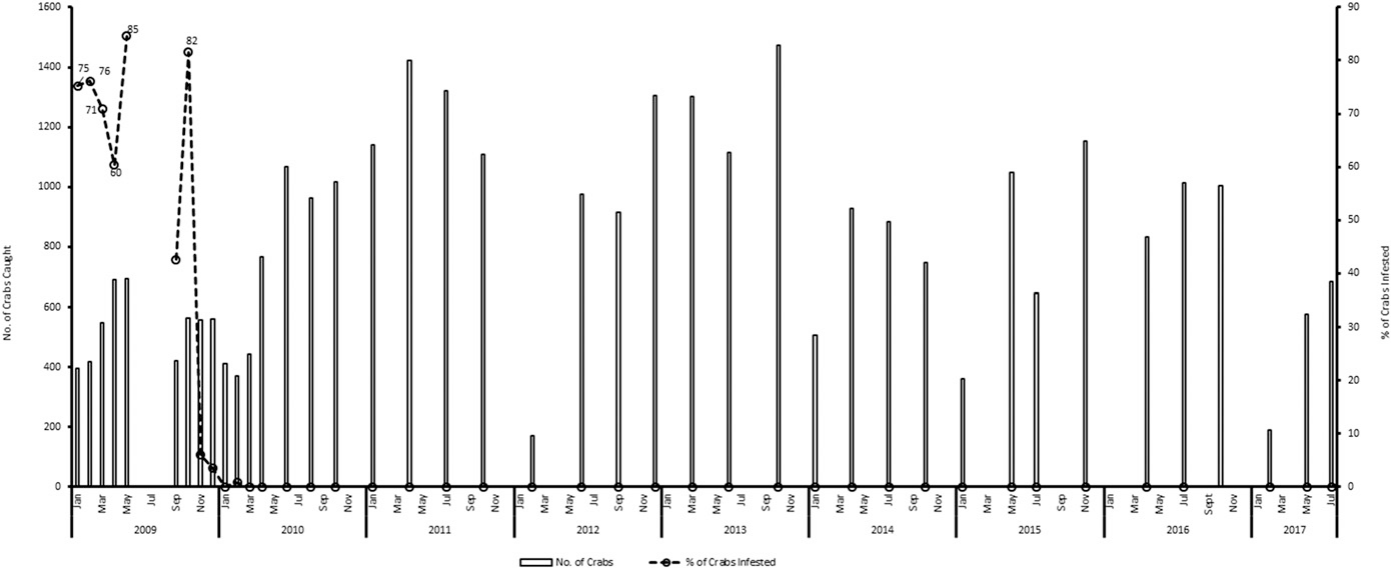
Trend of crab infestation (2009–2017).

Crab infestations were reduced to zero when larviciding was applied. The last larval stage of *S. neavei* on a crab was observed in February 2010. No other larvae were seen throughout 2013, when the transmission was declared interrupted (Figure 7). During the PTS period, none of 10,578 crabs were found to be carrying fly larvae (Table 2). Therefore, from March 2010 to July 2017, a period of at least 7 years, no infested crabs were observed (Figure 7).

**Table 2:** Number of crabs caught and assessed for infestation with aquatic stages of *Simulium neavei* during the posttreatment surveillance period (2014–2017)

Year	Quarterly visits	No. of sites monitored	Crabs caught	Crabs with aquatic stages of *Simulium neavei*
2014	4	11	3,066	0
2015	4	11	3,209	0
2016	3	11	2,852	0
2017	3	11	1,451	0
	Total		10,578	0

#### Serological results

In 2008, DBS samples from 2,796 children younger than 10 years were collected and tested with OV16 ELISA. A total of 44 (1.6%; 95% CI: 1.16–2.12) were OV16 positive (Table 3). In 2013, DBS samples of 2,978 children were examined; one child was OV16 positive (0.0%; 95% CI: 0–0.21). All the 3,079 DBS samples collected in early 2017 after a 3-year PTS period were negative for OV16 (95% CI: 0–0.16).

**Table 3:** Comparing OV16 results from Wambabya–Rwamarongo between 2008 and 2013

2008				2013			
Parish name	Number screened	Postive IgG4	Percent positive (95% CI)	Parish name	Number screened	Positive IgG4	Percent positive (95% CI)
Birungi	176	0	0 (0–0.021)	Birungu	144	0	0 (0–0.0324)
Budaka	46	1	2.2 (0.001–0.013)	Budaka	113	0	0 (0–0.035)
Bulimya	70	0	1.4 (0–0.088)				
Bwikya	196	0	0 (0–0.024)	Bwikya	495	1	0.2 (0–0.013)
Kabale	436	15	3.4 (0.020–0.057)	Kabale	770	0	0 (0–0.006)
Kaseeta	387	3	0.8 (0.002–0.025)	Kaseeta	260	0	0 (0–0.018)
Katanga	352	6	1.7 (0.008–0.039)	Katanga	374	0	0 (0–0.013)
Kibanjwa	60	1	1.7 (0–1.101)	Kibanjwa	139	0	0 (0–0.034)
Kidoma	486	7	1.4 (0.006–0.031)	Kidoma	416	0	0 (0–0.011)
Nyakabingo	158	9	5.7 (0.028–0.11)	Nyakabingo	267	0	0 (0–0.018)
Ruguse	429	2	0.5 (0–0.018)	Ruguse	170	0	0 (0–0.028)
Total	2,796	44	1.6 (0.012–0.021)	Total	2,978	1	0.03 (0–0.0021)

## DISCUSSION

The absence of *S. neavei* observed after a short period of larviciding with Abate^®^ indicated that transmission had been eliminated. The OV16 result of 2,978 DBS samples from children younger than 10 years in 2013 when interruption of transmission was declared, and 3,079 DBS samples from the same age-group in 2017 after 3-year posttreatment surveillance, provided additional evidence confirming the elimination of onchocerciasis in the Wambabya–Rwamarongo focus. Interestingly, when *S. neavei* was eliminated, *S. adersi* appeared to replace it. PCR results for *Simulium adersi* collected during the PTS periods were negative, strengthening our belief that elimination had been attained. All available evidence from Cameroon, Uganda, and Tanzania suggest that *S. adersi* is not a vector of onchocerciasis.^22^

Initially on conducting the larviciding of the rivers, there was some concern that migration of *S. neavei* from the Budongo focus might reestablish the vector population. Fortunately, no evidence for reinvasion was seen in the seven-year period from 2010 to 2017. Moreover, in 2018, *S. neavei*–transmitted onchocerciasis in the Budongo focus was declared interrupted by the UOEEAC, and the focus was moved to a 3-year PTS period.

OV16 results among children younger than 10 years residing close to the Bugoma Forest, southwest of the focus, were negative, showing no exposure to *O. volvulus*, and fly collection points within 6 km from the Bugoma Forest were negative for *S. neavei* adult flies. Crab-monitoring sites in the same area did not show any trace of infested crabs. There is a possibility that the Wambabya–Rwamarongo area may receive individuals with onchocerciasis who are displaced by political unrest in the nearby Democratic Republic of Congo. However, the possibility of enabling transmission within the focus is remote, given that the vector has been eliminated.

The absence of adult flies, infested crabs, and OV16-positive children for over 7 years after stopping intervention convinced the UOEEAC meeting in August 2017 that the Wambabya–Rwamarongo onchocerciasis focus was indeed isolated and there was no possibility of the focus being reseeded with the vectors of onchocerciasis. The focus was thus reclassified as “eliminated.” In August 2017, Wambabya–Rwamarongo joined other six onchocerciasis foci in Uganda (Victoria Nile, Itwala, Mbamba–Nkusi, Mt. Elgon, Imaramagambo, and Kashoya–Kitomi) in which onchocerciasis has been declared eliminated (Figure 2).

## CONCLUSION

The rapid interruption of transmission in the Wambabya–Rwamarongo focus was further evidence supporting the use of an aggressive complementary approach, applying vector control along with twice per year treatment with ivermectin to eliminate onchocerciasis. This approach demonstrated that onchocerciasis elimination is possible in Uganda and many foci from other onchocerciasis-endemic countries in Africa. Where vector elimination is not feasible, it is possible that targeted vector control can be applied to complement twice per year treatment with ivermectin. This complementary approach may substantially reduce the time necessary to interrupt *O. volvulus* transmission when compared with the time needed to interrupt transmission by using ivermectin alone,^23^ accelerating the push to continent-wide elimination.

## References

[1] Brown AW, 1962 A survey of *Simulium* control in africa. Bull World Health Organ 27: 511–527. 14015908 PMC2555867

[2] McMahon JP, 1967 A review of the control of *Simulium* vectors of onchocerciasis, Bull World Health Organ 37: 415–430. 5301384 PMC2554260

[3] Colbourne MJ Crosskey RW, 1965 Onchocerciasis and its Control in Uganda. AFR/Oncho/8 Rev.1, WHO/Oncho/30.65 Rev.1. Geneva, Switzerland: World Health Organisation.

[4] Prentice MA, 1974 *Simulium* control program in Uganda, 1974. Research and Control of Onchocerciasis in the Western Hemisphere. Washington, DC: Pan American Health Organization, Science Publication No. 298, 87–93.

[5] Raybould JN White GB, 1979 The distribution, bionomics and control of onchocerciasis vectors (Diptera: Simuliidae) in east Africa and the Yemen. Tropenmed Parasitol 30: 505–547. 538821

[6] Ayele T Walsh JF, 1991 Onchocerciasis Control in Uganda. WHO Mimeographed Document Geneva, Switzerland: World Health Organization.

[7] Walsh JF Garms R Lakwo T, 1996 Planning of Focal Vector Eradication in Onchocerciasis Foci in Uganda. Report to UNDP/World Bank/WHO Special Programme for Research & Training in Tropical Diseases (TDR)- (ID No.960012, Allotment No. GL/RES/TDR/045/FA/96/B) Geneva, Switzerland: Special Programme for Research & Training in Tropical Diseases (TDR), World Health Organization.

[8] Uganda Report, 2011 Guidelines for Certification of Onchocerciasis Elimination in Uganda. Kampala, Uganda: Uganda Government, Ministry of Health.

[9] Katabarwa MN 2018 After 70 years of fighting an age-old scourge, onchocerciasis in Uganda, the end is in sight. Int Health 10 (Suppl_1): i79–i88. 29471335 10.1093/inthealth/ihx044

[10] World Health Organisation, 1989 Resolutions and Decisions 1. The Forty-Second World Health Assembly, WHA42.31 (Thirteen plenary meeting, 19 May 1989, Hbk Res., Vol. II (1985), 1.16.2 Geneva, Switzerland: World Health Organization.

[11] Okello DO Ovuga EB Ogwal-Okeng JW, 1995 Dermatological problems of onchocerciasis in Nebbi district, Uganda. East Afr Med J 72: 295–298. 7555885

[12] Ovuga EB Okello DO Ogwal-Okeng JW Orwotho N Opoka RO, 1995 Social and psychological aspects of onchocercal skin disease in Nebbi district, Uganda. East Afr Med J 72: 449–453. 7498028

[13] Brieger WR 1998 The effects of ivermectin on onchocercal skin disease and severe itching: results of a multicentre trial. Trop Med Int Health 3: 951–961. 9892280 10.1046/j.1365-3156.1998.00339.x

[14] WHO, 2016 Guidelines for Stopping Mass Drug Administration and Verifying Elimination of Human Onchocerciasis: Criteria and Procedures. Geneva, Switzerland: World Health Organization.

[15] Katabarwa NM 2020 The Galabat-Metema cross-border onchocerciasis focus: the first coordinated interruption of onchocerciasis transmission in Africa. PLoS Negl Trop Dis 14: e0007830. 32027648 10.1371/journal.pntd.0007830 PMC7004312

[16] Mpagi J Katamanywa J Garms R, 2000 Dispersal range of *Simulium neavei* in an onchocerciasis focus of western Uganda. Med Vet Entomol 14: 95–99. 10759318 10.1046/j.1365-2915.2000.00221.x

[17] Barnley GR, 1975 Onchocerciasis. Uganda Atlas of Disease Distribution. Nairobi, Kenya: East African Publishing House, 38–40.

[18] Ndyomugyenyi R Lakwo T Habomugisha P Male B, 2007 Progress towards the elimination of onchocerciasis as a public-health problem in Uganda: opportunities, challenges and the way forward. Ann Trop Med Parasitol;101: 323–333. 17524247 10.1179/136485907X176355

[19] Garms R Lakwo TL Ndyomugyenyi R Kipp W Rubaale T Tukesiga E Katamanywa J Post RJ Amazigo UV, 2009 The elimination of the vector *Simulium neavei* from the Itwara onchocerciasis focus in Uganda by ground larviciding. Acta Trop 111: 203–210. 19446785 10.1016/j.actatropica.2009.04.001

[20] Katabarwa M 2014 Transmission of *Onchocerca volvulus* by *Simulium neavei* in Mount Elgon focus of eastern Uganda has been interrupted. Am J Trop Med Hyg 90: 1159–1166. 24686740 10.4269/ajtmh.13-0501 PMC4047747

[21] Richards FO 2018 Operational performance of the *Onchocerca volvulus* ‘OEPA’ Ov16 ELISA serological assay in mapping, guiding decisions to stop mass drug administration, and posttreatment surveillance surveys. Am J Trop Med Hyg 99: 749–752. 30014821 10.4269/ajtmh.18-0341 PMC6169192

[22] Hendy A 2018 The black fly vectors and transmission of *onchocerca volvulus* in Mahenge, south eastern Tanzania. Acta Trop 181: 50–59. 29410302 10.1016/j.actatropica.2018.01.009

[23] Smith ME 2019 Accelerating river blindness elimination by supplementing MDA with a vegetation “slash and clear” vector control strategy: a data-driven modeling analysis. Sci Rep 9: 15274. 31649285 10.1038/s41598-019-51835-0 PMC6813336

